# 3D microdevices that perform sample purification and multiplex qRT-PCR for early cancer detection with confirmation of specific RNAs

**DOI:** 10.1038/s41598-018-35772-y

**Published:** 2018-11-30

**Authors:** Yusuke Kimura, Masashi Ikeuchi, Yoshinori Inoue, Koji Ikuta

**Affiliations:** The University of Tokyo, 7-3-1 Hongo, Bunkyo-ku, Tokyo Japan

## Abstract

MicroRNA expression analysis is an important screening tool for the early detection of cancer. In this study, we developed two portable three-dimensional microdevices for multiple singleplex RNA expression analysis by microRNA purification and qRT-PCR as a prototype for point-of-care testing. These microdevices are composed of several types of modules termed ‘chemical IC chips’. We successfully reduced the heating area and fluorescence observation area, reduced the energy required for the reaction, and improved the portability of all systems in the devices. The purification microdevice could purify the microRNA from the sample using the FTA elute card system. The disposable reactor module mounted on both devices was easily fabricated by deforming a 100-μm-thick polypropylene film using an uncomplicated procedure. The qRT-PCR microdevice could perform reactions for samples of small volume. We purified microRNA from the HepG2 liver cancer cell line using the purification microdevice and confirmed the expression level of miR-224, which is a potential biomarker for liver cancer. Furthermore, we observed an increase in the fluorescence intensity when we performed qRT-PCR in the qRT-PCR microdevice. Therefore, the two developed microdevices show promise as a new portable tool for early cancer detection.

## Introduction

Point-of-care testing (POCT) is an important concept in the medical field with two main advantages. First, it allows rapid diagnosis, resulting in the early detection of diseases and improvement of survival rates. Second, tests can be performed in various places, increasing the opportunities for patient testing. Many patients show no symptoms in the early phases of disease. As a result, they rarely seek medical care, reducing the early detection of diseases. Therefore, POCT has the potential to confirm disease diagnosis in the early stages of various diseases that may be difficult to cure in advanced stages, the point at which conventional testing can identify the disease.

One of the most important applications of the POCT strategy is cancer diagnosis. Early detection has a major impact on the potential for complete recovery from all types of cancer. As is well known, the 5-year survival rate of cancer patients is significantly decreased when diagnosed at a later stage^1^. Although early detection methods have been developed for specific types of cancers, there is a general lack of early detection methods for many cancers.

Aberrant expression of specific RNAs occurs in the early phase of cancer. The abundance of microRNAs (miRNAs), which play important roles in gene regulation^2–4^, in exosomes in the blood has been shown to increase in the early stage of cancer development^5,6^. Therefore, exosomes can induce tumour progression and cancer metastasis^7,8^. Furthermore, the specific miRNAs showing aberrant expression depend on the tissue in which the primary tumour develops^9,10^. These observations indicate that miRNA expression analysis could enable cancer detection at an early phase and facilitate identification of the cancerous tissue.

For the detection of exosomal miRNA in the blood, we need to perform two procedures: exosomal miRNA purification from the blood and miRNA expression analysis. Although these procedures can be performed using conventional devices—ultracentrifugation for purification and thermal cycling for expression analysis—there are two main obstacles associated with their application in early cancer diagnosis. First, conventional devices require expensive reagents, resulting in a high medical cost for the patients or public health system. Second, conventional devices are generally too heavy and large for portable use. Given that patients in the early stages of cancer often show few to no symptoms, most patients will not seek medical care from specialised institutions that have such equipment available. These factors restrict the types of tests performed, thereby limiting the potential for early cancer detection.

Some microdevices can perform gene expression analysis using small volumes of reagents. Kopp *et al*.^11^ developed a polymerase chain reaction (PCR) device that can control the reaction by moving the reagent in the channel through the temperature zone required. Angione *et al*.^12^ performed real-time PCR in a droplet of mineral oil encompassing the reagents. Furthermore, Zhu *et al*.^13^ fabricated a multiplex quantitative reverse-transcription PCR (qRT-PCR) device that can be used with reagents at the sub-microlitre scale by applying droplet reagents into the oil pool on the device. Although these microdevices are useful, they still require a large control system, or the reactions are performed on a conventional thermal cycler. Tachibana *et al*.^14^ improved the channel device by moving the reagent only via capillary action, allowing the complicated control system to be eliminated. Although this microdevice addresses the portability issue, it can handle only one reagent in the reaction at a time and therefore cannot be used for parallel analysis. For cancer screening, the expression levels of several types of miRNAs must be evaluated, and therefore, a device that allows only a single analysis is disadvantageous for early detection. In addition, almost all of the microdevices developed to date require a complicated microfabrication process. Accurate gene expression analysis for clinical application requires a low-cost and disposable reactor to prevent any non-specific reactions from interfering with the clinical specimen. Furthermore, these microdevices do not purify the RNA from the sample—RNA purification must be performed on a large conventional device independently.

**Figure 1:**
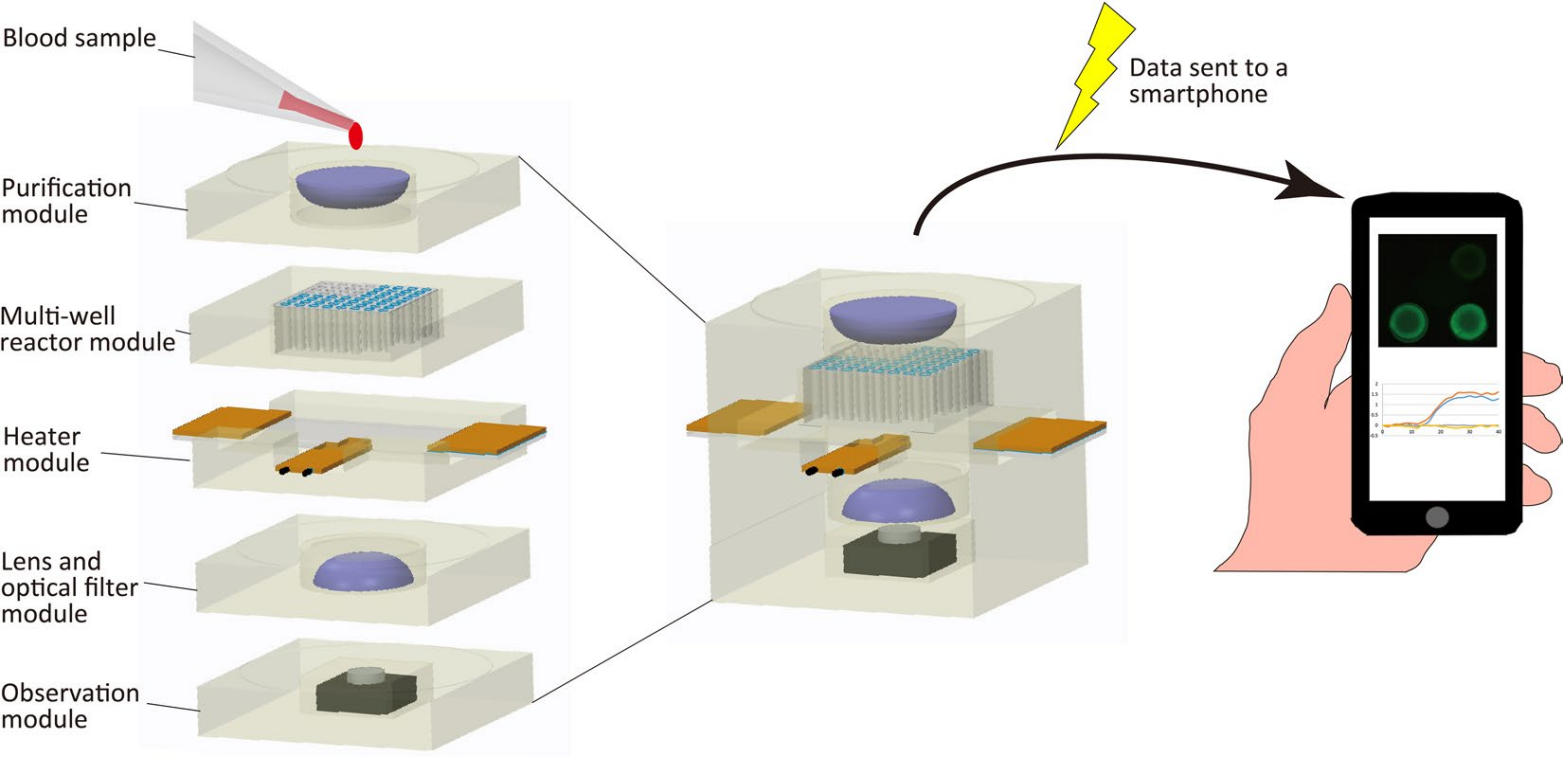
Conceptual model of the portable microdevice for early detection of cancer.

To address the limitations of current devices, we aimed to develop a simple, structured, portable microdevice that can perform miRNA purification and multiplex qRT-PCR. Figure 1 shows the conceptual model of the microdevice, which can automatically perform miRNA purification from the blood and multiple miRNA expression analysis by simple application of a blood sample. The data are quickly analysed and sent to a smartphone, on which the results can be easily evaluated. Offering portability and easy operation, this microdevice could be used to detect various types of cancer at an early stage for all patients.

To successfully create this microdevice, we fabricated a microdevice that can perform multiple singleplex reverse-transcription reactions in 2015^15^. Although this device can perform accurate reverse transcription, it cannot perform PCR thermal cycling. In 2016, we developed a microdevice that can perform real-time PCR^16^. This device has improved thermal conductivity in the reactor and a fabricated photosensor module. However, this device can only perform one reaction at a time and analyse one sample per reaction. Furthermore, these devices cannot purify miRNA from the sample.

In this study, we developed two independent microdevices as a prototype. One microdevice can perform miRNA purification, and the other can perform multiple singleplex RNA expression analysis by qRT-PCR. These microdevices are composed of different steric modules which are necessary for reactions based on the chemical integrated circuit (chemical IC) chip concept^17,18^. With this 3D structure, we successfully reduced the heating area, observation area, and energy required for the reaction.

The reactor mounted on each device was easily fabricated by deforming a 100-μm polypropylene (PP) film in a mould, with the advantages of high heat conductivity, transparency, low cost, and disposability. Using the energy-reducing chemical IC chip system concept, these prototype devices allow miRNA purification and multiple singleplex qRT-PCR reactions to be run using only the energy supplied from a battery. All the samples in the qRT-PCR microdevice can be observed simultaneously by a CMOS image sensor under the microdevice. Temperature, excitation light, and fluorescence intensity observation on the devices are controlled from a laptop computer (laptop). Thus, all the systems on the microdevices can be controlled from the laptop. Using these microdevices, we performed a complete reaction from miRNA purification to analysis of the miRNA expression level. We purified miRNA from the HepG2 liver cancer cell line and confirmed the expression level of miR-224, a potential biomarker for liver cancer^19,20^. In addition, we confirmed the increase in the fluorescence intensity of samples in the reactor when we performed qRT-PCR in the microdevice. Importantly, our microdevice offers the same sensitivity as the conventional device in spite of the small sample reaction. Therefore, this study may help determine whether the microdevices are suitable for practical use.

## Results

### Structure of the qRT-PCR microdevice

**Figure 2:**
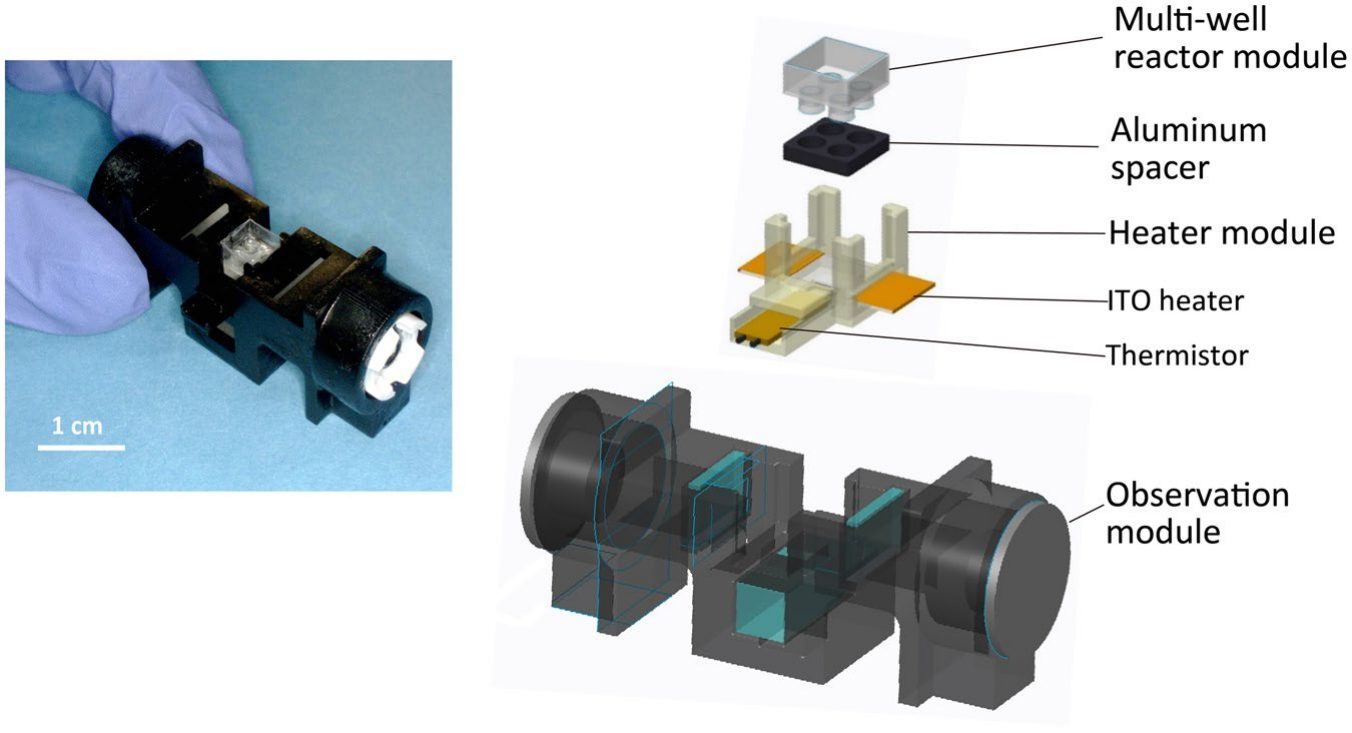
Overview of the prototype microdevice for qRT-PCR.

Figure 2 shows an overview of the prototype qRT-PCR microdevice. The device is hand-held and approximately 5.5 cm long, 2.5 cm wide, and 2.5 cm high, but it can perform all of the processes required for qRT-PCR.

**Figure 3:**
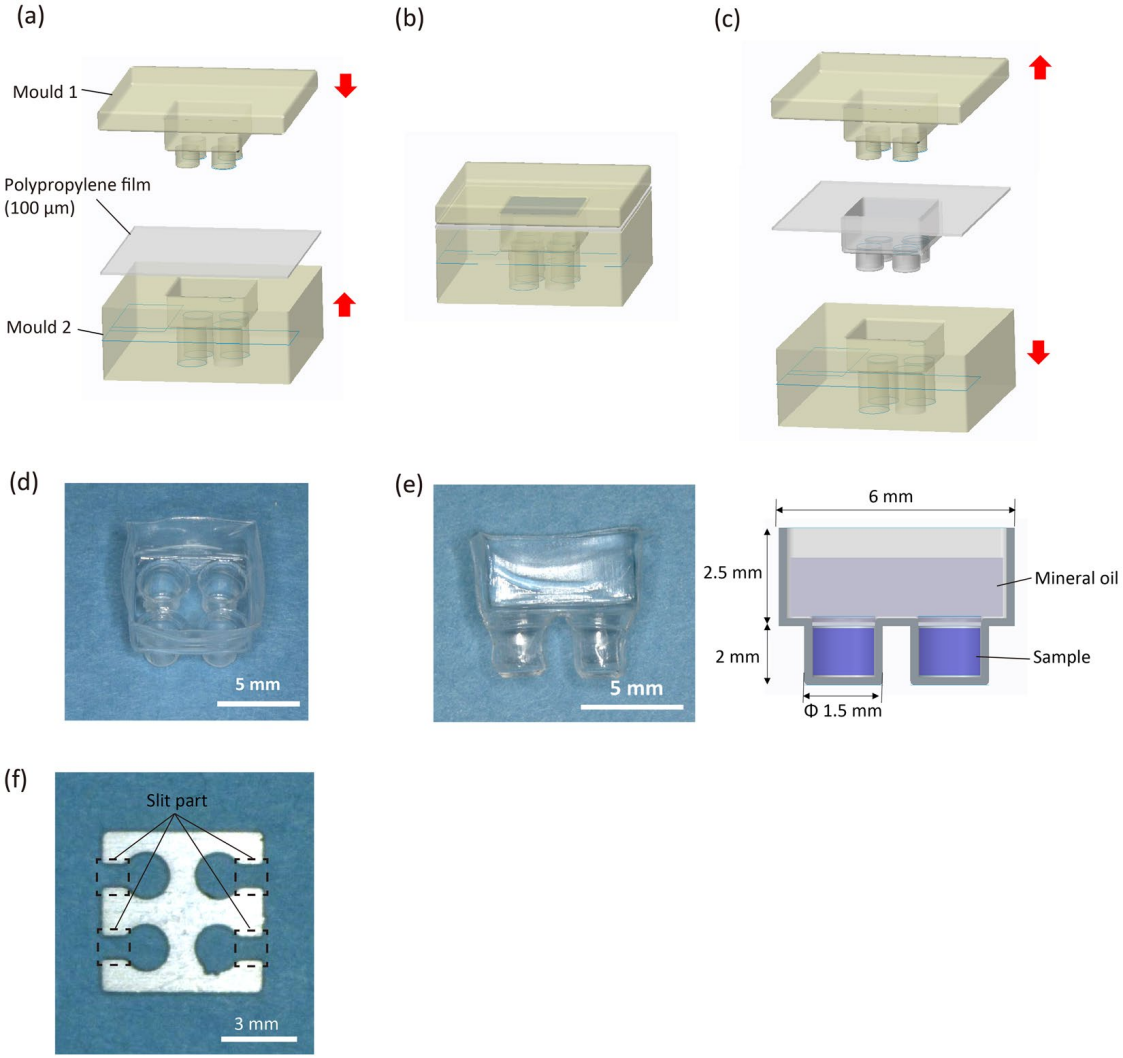
Polypropylene (PP) film multi-well reactor module. (**a**) Fabrication process for the PP film multi-well reactor module. The reactor was fabricated by placing the PP film between the moulds. (**b**) The PP film was heated to 120 °C for 1 h and deformed to the moulds. (**c**) The film cooled to room temperature, and the moulds were removed. (**d**) Fabricated PP film reactor. (**e**) Cross-section of the reactor. The sample is added to the well, and mineral oil is applied to the upper portion of the well to prevent evaporation. (**f**) Fabricated aluminium spacer.

Figure 3d shows the final fabricated PP film reactor, and Fig. 3e shows a cross-section of the reactor. The reactor has 4 wells and therefore can perform the analyses in parallel using multiple reagents or for different genes. Each well is φ1.5 mm and 2 mm deep and can hold a maximum of 3.5 µl of reagent. In the upper portion of the well, mineral oil can be added to prevent evaporation of the reaction reagents (Fig. 3e). The film reactor has a thinner wall than a conventional PCR tube, which can improve the thermal conductivity and transparency. In addition, the reactor is easily fabricated, and the cost of the PP film for fabricating a single reactor is under 30 cents. Thus, a disposable multi-well reactor can be easily fabricated for a low cost.

The aluminium spacer covers the area around the well except for the slit part, which allows the ITO heater to heat almost all sides of the well to increase the thermal conductivity (Fig. 3f). The excitation light is irradiated to each wells through the slit part. At this time, all sides of the well are covered by the spacer except for the slit part. This divides each wells clearly and prevents the optical crosstalk.

### Temperature control

**Figure 4:**
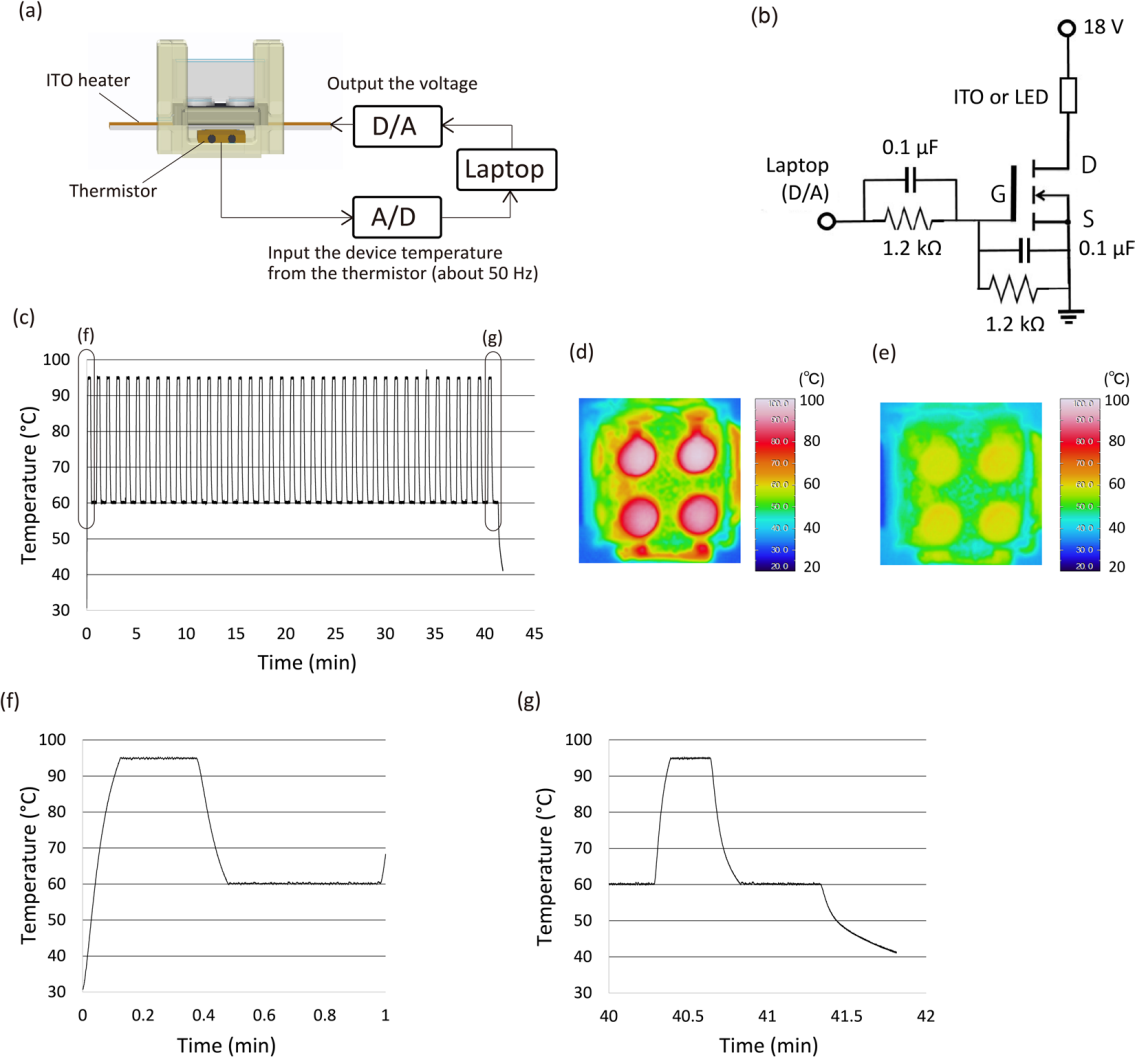
Device temperature control. (**a**) Temperature control system of the microdevice. The device temperature was calculated from the resistance of the thermistor by the laptop. When the device temperature falls below the preset temperature, the signal is outputted, and the ITO heats up. (**b**) ON/OFF control circuit. We used two 9 V batteries (18 V) as the power supply. We output the ON/OFF signal from the laptop and controlled the voltage output by inputting the ON/OFF signal to the MOSFET gate. This circuit was also used to control the ON/OFF switch of the LEDs (Supplementary Fig. 4). (**c**) Temperature change over time calculated from the resistance of the thermistor during PCR thermal cycling. (**d**,**e**) Device temperature monitored by thermography at each PCR thermal phase. (**f**,**g**) Closer view of (**c**) for the 1^st^ and 40^th^ PCR thermal cycles.

As shown in Fig. 4, effective PCR temperature control was achieved on the prototype microdevice. Figure 4c shows the device temperature calculated from the resistance of the thermistor when we performed PCR thermal cycling with the empty reactor. As indicated by the graph, the device was accurately controlled to the preset temperature throughout PCR. Also the reactor temperature was accurately controlled in all wells of the device at the denaturation phase (95 °C) and extension phase (60 °C) (Fig. 4d,e). Comparison of the 1^st^ cycle with the 40^th^ cycle revealed good maintenance of the temperature throughout PCR (Fig. 4f,g).

### Structure of the miRNA purification microdevice

**Figure 5:**
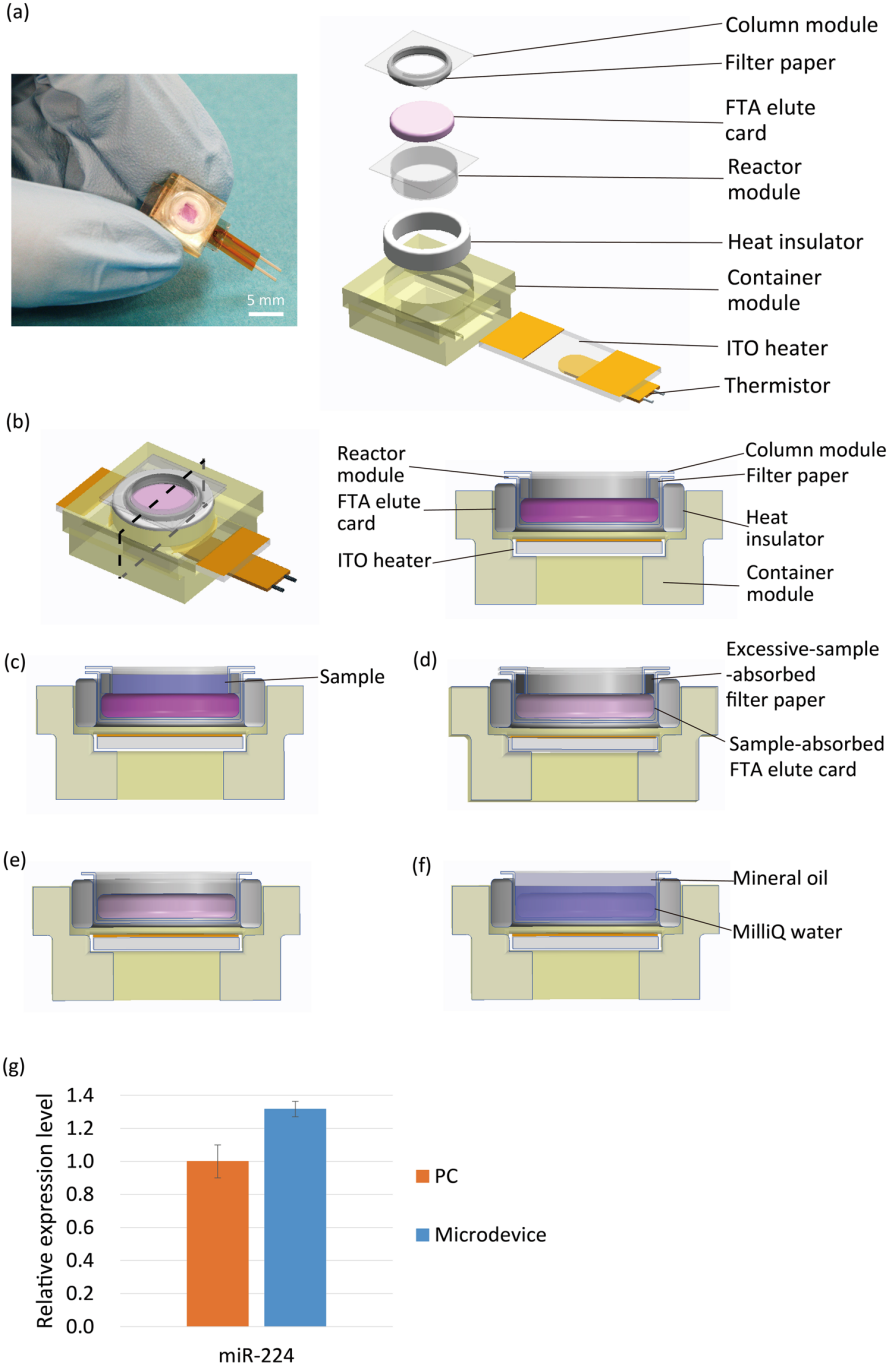
Purification microdevice. (**a**) Overview of the prototype microdevice for miRNA purification. (**b**) Cross-section of the microdevice. (**c**–**f**) Protocol of the purification microdevice. (**c**) First, sample is applied to the column module. (**d**) Then, the sample is absorbed into the FTA elute card on the reactor module. Excessive sample is absorbed by the filter paper. After drying the FTA elute card, (**e**) the column module is ejected, and (**f**) MilliQ water and mineral oil are applied to the reactor module. By heating the device via the ITO heater at 98 °C for 30 min, the purified miRNA was released from the FTA elute card and total miRNA is purified. (**g**) Expression level of miR-224 purified on the purification microdevice. The PC was generated by purification using the FTA elute card according to the manufacturer’s instructions. The graph shows the relative expression level of miR-224. The error bars represent standard deviation.

Figure 5a,b shows the miRNA purification microdevice. The device is also hand-held and approximately 1.4 cm long, 1.2 cm wide, and 0.7 cm high, but it can perform miRNA purification from a biospecimen sample. For the miRNA purification, we used the FTA elute card system (GE healthcare). This card can denature the protein and bind nucleic acids on the fibre structure. Previous studies have purified miRNA from serum or cells using the FTA elute card^21,22^. The contact part of the container module and reactor module were covered by a heat insulator (BSFP300, SAKAGUCHI E.H VOC CORP.), which improved the thermal conductivity between the ITO heater and reactor module. The ITO heater was cut to 6 × 18 mm, and both ends of the 6 × 6-mm were metal deposited. The purification microdevice used the same temperature control system as the qRT-PCR microdevice. Figure 5c–f details the protocol of the purification microdevice. Mineral oil was applied to prevent evaporation of the MilliQ water. At the end of the procedure, the purified miRNA was released from the FTA elute card.

### miRNA purification

Figure 5g shows the result of the purification performed by the purification microdevice. In this experiment, miRNA was purified from 1.0 × 10^5^ cells/30 µl HepG2 cell suspension, and the expression level of miR-224 was analysed. The positive control (PC) was generated by performing the purification with a conventional device using the FTA elute card. The error bars represent standard deviation. Each post-purification sample was subjected to qRT-PCR on the conventional device (Eco, Illumina), and the expression level of miR-224 was compared between the two techniques. As shown in Fig. 5g, the purification microdevice can perform miRNA purification with results comparable to those of the conventional method.

### qRT-PCR

**Figure 6:**
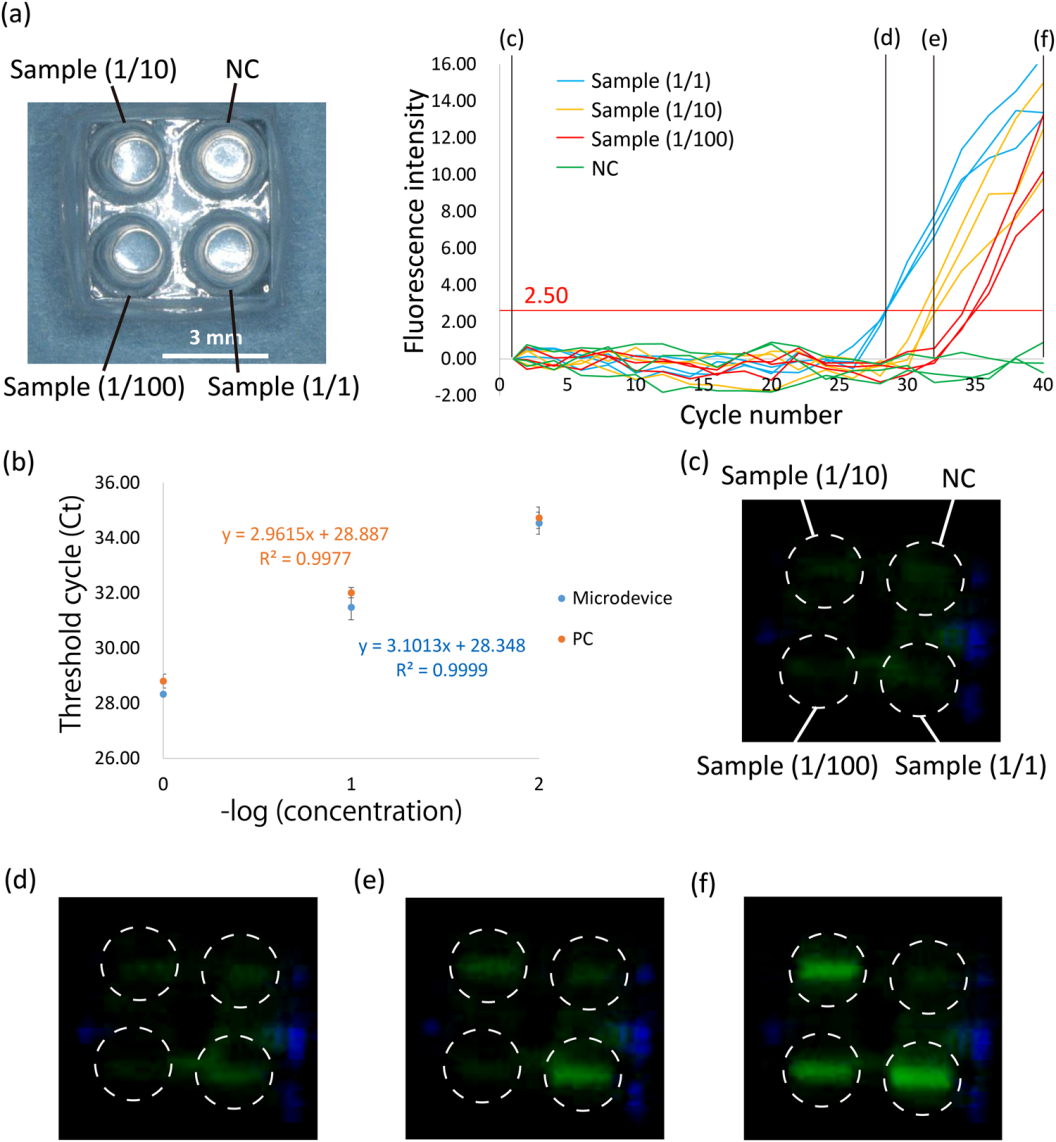
qRT-PCR on the microdevice. (**a**) Increase in the fluorescence intensity per PCR thermal cycle. We applied samples that were diluted 1/1, 1/10, or 1/100 (final RNA concentration: 5 ng/μl, 0.5 ng/μl, or 50 pg/μl) or MilliQ water as the NC. The sample was purified from the HepG2 cell line using the purification microdevice, and the expression level of miR-224 was analysed. The red line shows the fluorescence threshold line (=2.50). (**b**) Real-time PCR standard curve extracted from results in (**a**) showing the Ct as a function of –Log (cDNA concentration). PC represents the results obtained when we performed the same experiment on the conventional device. The fluorescence threshold line of PC was 0.07. The PC results are shown in Supplementary Fig. 1. The error bars represent standard deviation. (**c**–**f**) Images captured by the CMOS image sensor at the 1^st^, 28^th^, 32^nd^, or 40^th^ PCR thermal cycle, respectively. The white dotted lines shows the position of wells.

qRT-PCR was performed on the fabricated microdevice to confirm the expression level of miR-224 using the miRNA purified from the HepG2 cell suspension by the purification microdevice (Fig. 6). Figure 6a shows the fluorescence intensity of each well based on the PCR thermal cycle number. The fluorescence intensity was analysed using ImageJ software (National Institutes of Health), and the average fluorescence intensity of each well was calculated. We performed the same experiments for 3 times. The graph shows the fluorescence intensity after subtracting that of the 1^st^ cycle in each well to confirm the increase in the fluorescence intensity.

We applied the 200 nl/well reverse transcription sample that included the purified sample diluted to 1/1, 1/10, or 1/100 (final total RNA concentration: 5 ng/μl, 0.5 ng/µl, or 50 pg/µl) or MilliQ water as the negative control (NC) and performed reverse transcription. After that, we applied 1.8 µl PCR mix to each well and performed real-time PCR. As shown in Fig. 6a, we confirmed the increase in fluorescence intensity from each well according to the purified sample concentration. To confirm the accuracy of the PCR performed in the microdevice, we extracted the standard real-time PCR curve from the results in Fig. 6a, showing the threshold cycle (Ct) as a function of –Log (cDNA concentration) (Fig. 6b). The fluorescence threshold line was 2.50. The error bars represent standard deviation. As shown in Supplementary Fig. 1, PC represents the result when we performed the same experiment on the conventional device (Eco, Illumina); the fluorescence threshold line of PC was 0.07. As demonstrated in Fig. 6b, we confirmed a similar trend between PC and the microdevice. Supplementary Fig. 2 shows the average and the standard deviation of the fluorescence intensity based on the PCR thermal cycle number when we repeated the same experiment for 3 times on the microdevice or the conventional device. Although the standard deviation on the microdevice was larger than conventional device, we confirmed almost the same graph shape. These results suggested that our microdevice maintains the same detection range as the conventional device in spite of the small volume sample. Figure 6c–f shows the images captured by the CMOS sensor during the 1^st^, 28^th^, 32^nd^, and 40^th^ PCR cycles. We confirmed a significant increase in the fluorescence intensity by the naked eye according to the sample concentration. These results indicate that we succeeded in performing miRNA expression analysis—including miRNA purification—on the microdevices.

## Discussion

We have developed two portable microdevices that perform miRNA purification and multiple singleplex qRT-PCR as a prototype for the early detection of cancer. We observed a slight delay in the cooling phase from 95 °C to 60 °C at the 40^th^ cycle in the temperature control experiment (Fig. 4f,g). This is because the prototype device lacks its own cooling system, and therefore, the temperature of the entire device was higher at the 40^th^ cycle than at the 1^st^ cycle. Thus, more time was required to cool the device to the preset temperature.

In the purification experiment, the miR-224 expression level as determined by the purification microdevice was higher than that recorded by the conventional device. This is because MilliQ water was evaporated at the heating phase. Because of the fibre construction of the FTA elute card, an air bubble was trapped in the FTA elute card. And during the heating process, the bubble was released and the MilliQ water was evaporated with the bubble, effectively concentrating the purified sample. To perform accurate purification, we must completely prevent MilliQ water evaporation.

One purpose of the microdevice is the analysis of a small sample. When we performed the experiment with a small sample, sensitivity was degraded because of the small target gene volume and low fluorescence intensity. Supplementary Fig. 3 shows the transmittance of the excitation light and fluorescence through the 100-µm PP film and 350-µm PP sheet (the same thickness of a conventional PCR tube). The 100-µm PP film improved the transmittance by about 1.4 times compared with that of the 350-μm PP sheet. The maintenance of sensitivity demonstrated by our developed microdevice is attributed to this high transmittance of excitation and fluorescent light through the PP film.

In our experiment, the developed qRT-PCR microdevice quantified the specific miRNA volume from a sample containing 50 pg/µl total miRNA. The total miRNA concentration in the blood of a healthy person is about 70 pg/µl, and the total miRNA concentration increases to about 160 pg/µl in a lung cancer patient, as an example^23^. This suggests that our microdevice can quantify the miRNA volume even from the blood of healthy people, which contains a low concentration of miRNA. Thus, it is possible to detect miRNA cancer biomarkers at the early stage with our microdevice. However, we performed miRNA purification only from the cell suspension and did not use blood. Consequently, we need to perform miRNA purification from human blood and analyse the miRNA expression level to verify the clinical usefulness.

In this study, we successfully analysed a cancer-specific miRNA biomarker by performing miRNA purification and qRT-PCR using two independent microdevices. Nonetheless, we must transfer the sample from the purification microdevice to the qRT-PCR microdevice by hand and have not unified the two microdevices based on the chemical IC concept. We also have not yet created a system to analyse the results automatically and send on a smartphone as indicated in Fig. 1. When we fabricate the systems for sample transfer from the purification device to qRT-PCR device and data analysing and transmission, our microdevice will be able to perform cancer screening automatically at any location.

## Conclusion

In this study, we developed two POCT microdevices that can perform multiple RNA expression analyses by miRNA purification and qRT-PCR for the early detection of cancer. The purification device can purify miRNA from cell suspension samples using the FTA elute card system. The qRT-PCR device contains 4 wells and can simultaneously perform 4 sample analyses. These devices are also portable and include the entire system to control the devices. We anticipate that when we combine these two devices and create a system to analyse the results on a smartphone, our microdevice will be more practical for performing cancer analyses worldwide.

## Methods

### qRT-PCR microdevice fabrication

qRT-PCR microdevice consists of 4 parts: a multi-well reactor module to hold the sample and house the reaction, an aluminium spacer to improve the thermal conductivity to the samples, a heater module included in the ITO heater (0081, Geomatec) to heat the prototype device and a thermistor (104JT, Semitec) to monitor the temperature of the device, and an observation module to observe the fluorescence intensity of the samples.

Figure 3 illustrates the fabrication process for the multi-well reactor module. The multi-well reactor module was fabricated by deforming a 100-µm-thick PP film (General Science Corporation) in a mould. PP is used for the fabrication of conventional PCR tubes and does not interfere with the reaction. We fabricated a pair of moulds using micro-stereolithography with computer-aided design. Based on the design blueprint, the moulds were fabricated by hardening photocurable resin (SCR751, D-MEC) while irradiating with a He-Cd laser at micro-stereolithography machine (SCS-1000HD, D-MEC). The moulds were heated and hardened at 150 °C for 6 h to prevent deformation during the reactor fabrication process. By placing the PP film between the moulds (Fig. 3a) and heating to 120 °C for 1 h (Fig. 3b), the film was deformed to the shape of the moulds. The film cooled to room temperature, and the moulds were removed (Fig. 3c).

The aluminium spacer was fabricated by processing a 1.5-mm-thick aluminium board to the reactor size using a wire cutter (Fig. 3f).

The heater module was fabricated by micro-stereolithography. The ITO heater was cut to 6 × 14 mm, and both ends of the 6 × 4-mm metal deposited by the ion sputtering apparatus (E-1030, Hitachi) to improve the conductivity.

The observation module was fabricated by micro-stereolithography and painted with black-coloured spray (Creative life spray, Asahipen) to prevent reflected excitation light from passing through the photocurable resin and allow observation of the fluorescence intensity of the sample. Powerful LED lights were mounted to both ends of the module (OSB5XNE1C1E, OptoSupply) to produce the excitation light to irradiate samples through the low-pass filter (PMS490, Asahi Spectra), and a CMOS image sensor (BSW50KM02, BUFFALO) was mounted under the module to observe the fluorescent light of the sample through the bandpass filter (MZ0520, Asahi Spectra).

### Temperature control system

Figure 4a shows the temperature control system of the microdevice. The device is heated by an ITO heater positioned under the reactor module. The device temperature is controlled by monitoring the resistance of the thermistor under the ITO heater. The thermistor is a thermo sensor whose resistance is related to the temperature. The process temperature is controlled from a laptop. The device temperature is monitored at 50 Hz by measuring the resistance of the thermistor and calculating  from it on the laptop. Figure 4b shows the circuitry of the ON/OFF control system. We used two 9 V batteries (18 V) as the power supply. Using the Analog I/O PC Card (ADA16-8/2 (CB) L, CONTEC) on the laptop, we output the ON/OFF signal and control the output of the voltage from the batteries by inputting the ON/OFF signal to the gate of the MOSFET (IRF530A, Fairchild). The voltage of the batteries is outputted to the ITO heater when the monitored temperature falls below the preset temperature. The calculation and temperature control program were prepared with LabVIEW ver8.2 (National Instruments).

### Observation system

Supplementary Fig. 4 shows the method for visualisation of the fluorescence intensity using the observation module. The excitation light from both LEDs is directed to the samples in the reactor module through the low-pass filter. Then, the fluorescent light is irradiated from the samples in each well. The fluorescent light passes through the bandpass filter and is detected by the CMOS image sensor. The image captured by the CMOS image sensor is analysed by ImageJ software on the laptop. The optical axis is positioned at a right angle to prevent direct irradiation of the CMOS sensor with LED light, which may prevent to misdetection.

LED control is performed by the ON/OFF control circuit, which is the same design as the temperature control system (Fig. 4b). This circuit also uses two 9 V batteries as the power supply and is controlled by the same laptop. The LED control program was also prepared with LabVIEW ver8.2 and synchronised to the temperature control program.

### Temperature control experiment

The temperature of the qRT-PCR microdevice was maintained at 95 °C for 15 s and 60 °C for 30 s using the temperature control system. In this experiment, we used the empty reactor to measure the device temperature from the resistance of the thermistor. To confirm the actual temperature of the reactor, the reactor was observed by thermography (R300SR, Avio) at the denaturation phase (95 °C) and extension phase (60 °C) of the 1^st^ cycle (thermal emissivity: 0.94).

### miRNA purification microdevice fabrication

miRNA purification microdevice consists of 4 parts: a column module to receive the sample and reject excessive sample, a reactor module to perform the purification by FTA elute card, a container module to unify these modules, and an ITO heater and thermistor. The column module and reactor module were fabricated by the same process used for PP film reactor module fabrication as described above. The bottom of the column module contained a hole (φ3 mm), and the applied sample was absorbed by the FTA elute card (φ4 mm) on the reactor module through the hole. Filter paper was positioned around the column module to absorb the excessive sample that passed through the FTA elute card.

### Culture of HepG2 cell line

The HepG2 cell line was obtained from the RIKEN BRC Cell Bank, maintained in Dulbecco’s modified Eagle’s medium supplemented with 10% heat-inactivated foetal bovine serum, and incubated at 37 °C in humidified air with 5% CO_2_.

### miRNA purification

HepG2 cells (1.0 × 10^5^) were collected and suspended in 30 µl PBS(−). The cell suspension was applied to the column module of the purification microdevice, and through the hole of the column module, the suspension was applied to the FTA elute card (GE Healthcare) positioned on the reactor module. Then, the device was placed at room temperature for 1 h, and the FTA elute card was dried. After that, the column module was ejected, and 30 µl each of MilliQ water and mineral oil (Sigma) was applied. Next, the purification device was heated at 98 °C for 30 min by the temperature control system, and total miRNA was purified. The PC sample was also prepared from the same cell suspension. Total miRNA was purified using the FTA elute card according to the manufacturer’s instructions.

With each purified solution, qRT-PCR was performed. The reverse-transcription reagent was prepared by mixing 1 μl purified solution, 5 × has-miR-224 RT primer (TaqMan), 0.5 mM dNTPs (Invitrogen), 5 mM MgCl_2_ solution (Invitrogen), 10 × RT buffer (Invitrogen), 10 mM dithiothreitol (Invitrogen), 2 U/µl RNase inhibitor (Invitrogen), and 10 U/µl SuperScript III RT (Invitrogen) (total volume: 20 µl). Then, reverse transcription was performed by adjusting the device temperature to 50 °C for 50 min followed by 85 °C for 5 min with a conventional thermal cycler (T-100, Bio-Rad). After that, real-time PCR reagent was prepared by mixing 1 µl post-reverse-transcription solution, 2 × TaqMan Universal Master Mix, no UNG (TaqMan), and 20 × has-miR-224 probe and primer (TaqMan) (total volume: 30 µl). Finally, the reagent was applied to the 48-well plate (Illumina) at 10 µl/well, and real-time PCR was performed at 95 °C for 10-min incubation (1^st^ cycle only), 95 °C for 15 s, and 60 °C for 30 s for 40 cycles.

### qRT-PCR experiment

The reverse-transcription reagent was prepared by mixing 5, 0.5, or 0.05 ng/μl HepG2 cell total miRNA purified by the purification microdevice (the concentration of which was measured on a Qubit 3.0 fluorometer (Invitrogen)), 5 × has-miR-224 RT primer, 0.5 mM dNTPs, 5 mM MgCl_2_ solution, 10 × RT buffer, 10 mM dithiothreitol, 2 U/µl RNase inhibitor, and 10 U/µl SuperScript III RT. Then, 200 nl of reverse-transcription reagent was added to each well of the reactor using the Pipetman (P2, GILSON), and 20 µl of mineral oil (Sigma) was added to the upper portion of the well. Reverse transcription was performed by adjusting the device temperature to 50 °C for 50 min followed by 85 °C for 5 min.

For real-time PCR, 20 × has-miR-224 probe and primer and 2 × TaqMan Universal Master mix, no UNG, were mixed, and 1.8 µl of the mixture was added to each well using the Pipetman (P2, GILSON). Real-time PCR was then performed at 95 °C for 10-min incubation (1^st^ cycle only), 95 °C for 15 s, 60 °C for 30 s, and 60 °C for 1 s with LED irradiation and image capture for 40 cycles. The captured images were analysed using ImageJ software.

For the PC, qRT-PCR was performed in the same manner as the experiment described above but with the conventional device. Reverse transcription was performed using 20 µl reverse-transcription reagent with a conventional thermal cycler (T-100, Bio-Rad) under the same thermal conditions; 9 µl real-time PCR mixture was added to 1 µl of the post-reverse-transcription sample, and real-time PCR was performed by the conventional system (Eco, Illumina) using the same thermal conditions.

## Electronic supplementary material

Supplementary Information

## Data Availability

All data generated or analysed during this study are included in this published article (and its Supplementary Information files).
