# Supplementary material for: 3D microdevices that perform sample purification and multiplex qRT-PCR for early cancer detection with confirmation of specific RNAs
Source: Sci Rep. 2018 Nov 30;8:17480. doi: 10.1038/s41598-018-35772-y (PMC6269476; doi:10.1038/s41598-018-35772-y)
Supplement: Supplementary file 1 — Supplementary Information [file 41598_2018_35772_MOESM1_ESM.pdf]

Supplementary Information

3D microdevices that perform sample purification and multiplex qRT-PCR for early cancer detection with confirmation of specific RNAs

Yusuke Kimura, Masashi Ikeuchi, Yoshinori Inoue, and Koji Ikuta*

The University of Tokyo, 7-3-1 Hongo, Bunkyo-ku, Tokyo, JAPAN

*** Corresponding author**:
Koji Ikuta
Tel: +81-3-5452-5162
Fax: +81-3-5452-5163
Email: ikuta@rcast.u-tokyo.ac.jp

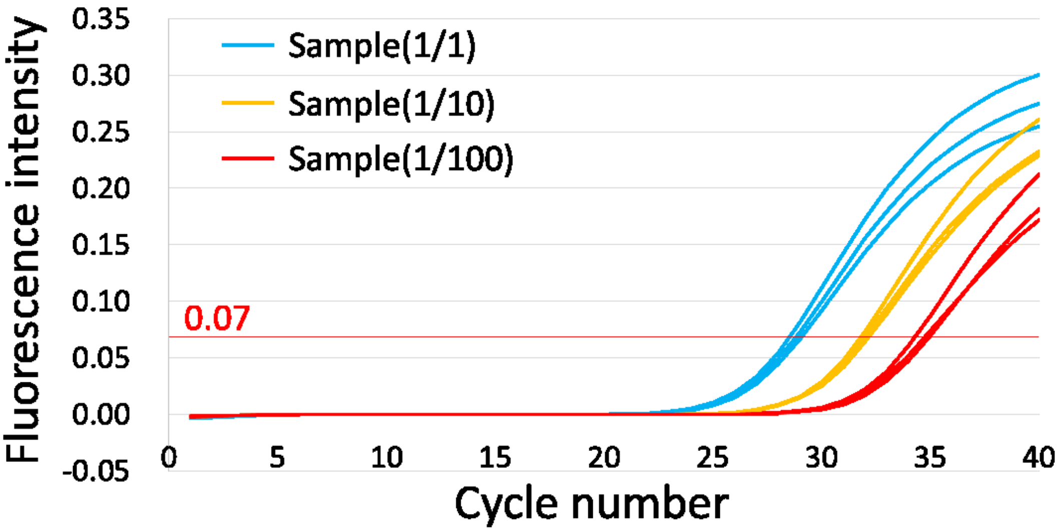

Supplementary Fig. 1 qRT-PCR on a conventional device. This is the result of qRT-PCR using the same sample as that used in Fig. 6a but on a conventional device (Eco, illumina). The red line shows the fluorescence threshold line (=0.07).

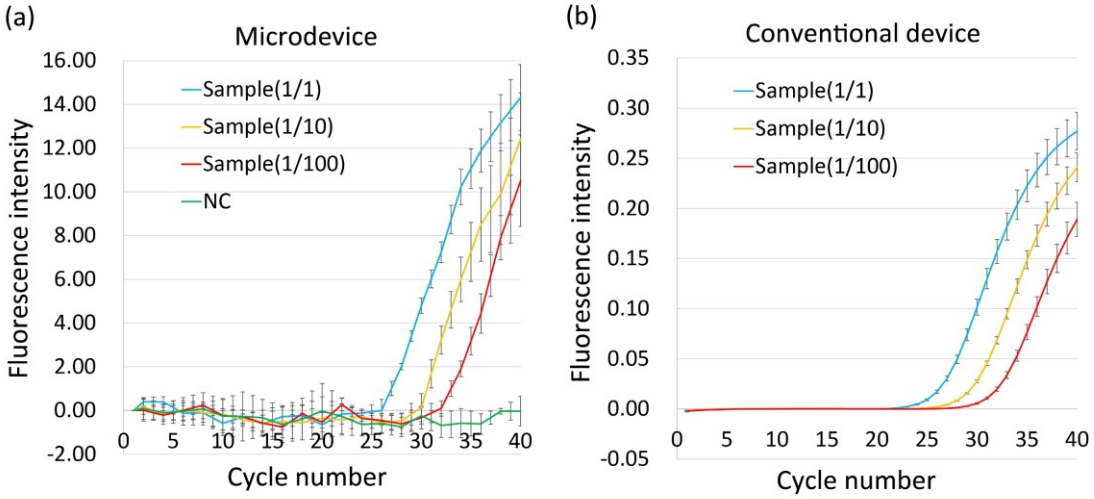

Supplementary Fig. 2 The average and the standard deviation of qRT-PCR. These graphs show the average and the standard deviation of the fluorescence intensity when we repeated the same qRT-PCR experiment for 3 times (a) on the microdevice or (b) on the conventional device.

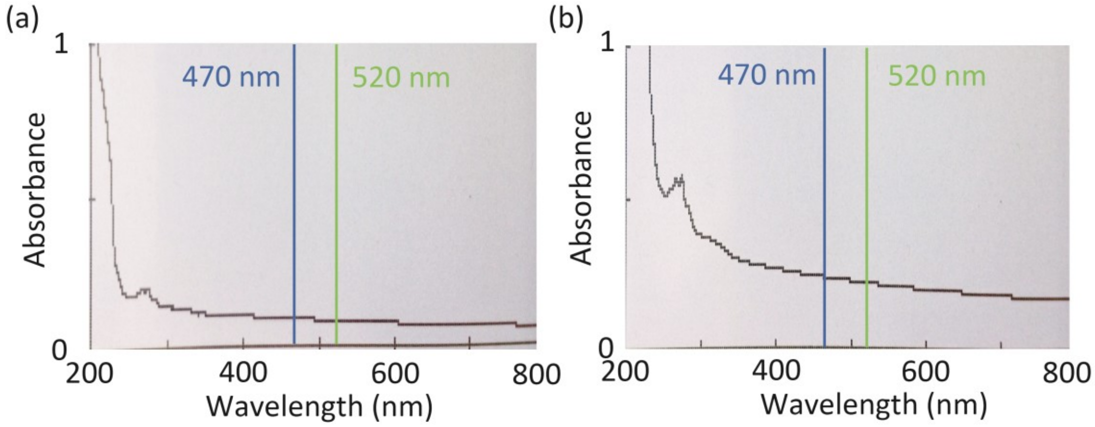

Supplementary Fig. 3 The absorbance of the (a) 100 µm PP film and (b) 350 µm PP sheet. We measured the absorbance of the 100 µm PP film and 350 µm PP sheet by the absorptiometer (UV-1800, SHIMADZU) and calculated the transmittance of the excitation light and fluorescence used in this paper.

100 µm PP film 470 nm: 82.4%, 520 nm: 83.6%

350 µm PP sheet 470 nm: 60.7%, 520 nm: 59.5%

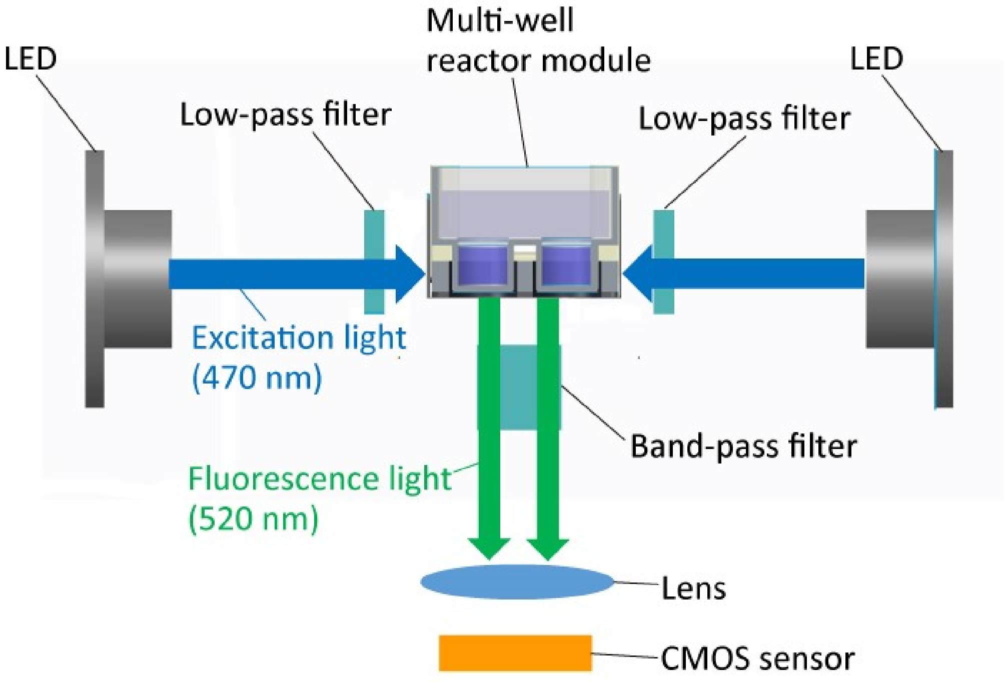

Supplementary Fig. 4 Observation system using the observation module.
